# A polymorph of diaqua­bis(pyrazine-2-carboxyl­ato-κ^2^ *N* ^1^,*O*)copper(II)

**DOI:** 10.1107/S1600536809045371

**Published:** 2009-11-04

**Authors:** Guan-Hua Wang, Ren-Ling He, Fan-Jin Meng, Ning-Hai Hu, Jing-Wei Xu

**Affiliations:** a The State Key Laboratory of Electroanalytical Chemistry, Changchun Institute of Applied Chemistry, Chinese Academy of Sciences, Changchun 130022, People’s Republic of China

## Abstract

The title compound, [Cu(C_5_H_3_N_2_O_2_)_2_(H_2_O)_2_], is a new polymorph of the previously reported compound [Klein *et al.* (1982 ▶). *Inorg. Chem.* **21**, 1891–1897]. The Cu^II^ atom, lying on an inversion center, is coordinated by two N atoms and two O atoms from two pyrazine-2-carboxyl­ate ligands and by two water mol­ecules in a distorted octa­hedral geometry with the water mol­ecules occupying the axial sites. Inter­molecular O—H⋯O, O—H⋯N and C—H⋯O hydrogen bonds connect the complex mol­ecules into a two-dimensional layer parallel to (10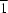), whereas the previously reported polymorph exhibits a three-dimensional hydrogen-bonded network.

## Related literature

For general background to metal complexes of pyrazine­carboxyl­ates, see: Dong *et al.* (2000 ▶); Kubota *et al.* (2006 ▶); Luo *et al.* (2004 ▶); Ptasiewicz-Bak *et al.* (1995 ▶). For the previously reported polymorph, see: Klein *et al.* (1982 ▶). For a related structure, see: Chutia *et al.* (2009 ▶).
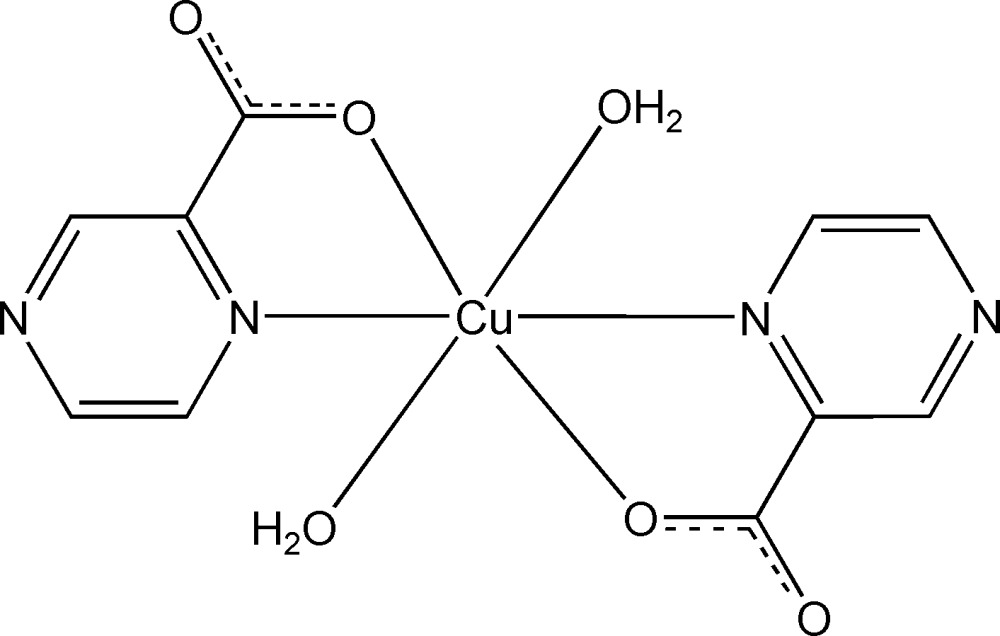


## Experimental

### 

#### Crystal data

[Cu(C_5_H_3_N_2_O_2_)_2_(H_2_O)_2_]
*M* *_r_* = 345.76
Monoclinic, 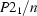

*a* = 6.7066 (12) Å
*b* = 7.9041 (14) Å
*c* = 12.030 (2) Å
β = 105.036 (2)°
*V* = 615.88 (19) Å^3^
*Z* = 2
Mo *K*α radiation
μ = 1.81 mm^−1^
*T* = 293 K
0.29 × 0.25 × 0.20 mm

#### Data collection

Bruker SMART APEX CCD diffractometer
Absorption correction: multi-scan (**SADABS**; Sheldrick, 1996 ▶) *T*_min_ = 0.626, *T*_max_ = 0.711
3322 measured reflections
1212 independent reflections
1119 reflections with *I* > 2σ(*I*)
*R*_int_ = 0.014

#### Refinement

*R*[*F* ^2^ > 2σ(*F* ^2^)] = 0.024
*wR*(*F* ^2^) = 0.071
*S* = 1.10
1212 reflections
98 parameters
H-atom parameters constrained
Δρ_max_ = 0.24 e Å^−3^
Δρ_min_ = −0.27 e Å^−3^

### 

Data collection: *SMART* (Bruker, 2007 ▶); cell refinement: *SAINT* (Bruker, 2007 ▶); data reduction: *SAINT*; program(s) used to solve structure: *SHELXS97* (Sheldrick, 2008 ▶); program(s) used to refine structure: *SHELXL97* (Sheldrick, 2008 ▶); molecular graphics: *SHELXTL* (Sheldrick, 2008 ▶) and *Mercury* (Macrae *et al.*, 2006 ▶); software used to prepare material for publication: *SHELXTL*.

## Supplementary Material

Crystal structure: contains datablocks global, I. DOI: 10.1107/S1600536809045371/is2480sup1.cif

Structure factors: contains datablocks I. DOI: 10.1107/S1600536809045371/is2480Isup2.hkl

Additional supplementary materials:  crystallographic information; 3D view; checkCIF report

## Figures and Tables

**Table 1:** Selected bond lengths (Å)

Cu1—O1	1.9486 (12)
Cu1—N1	1.9753 (14)
Cu1—O1*W*	2.6143 (14)

**Table 2:** Hydrogen-bond geometry (Å, °)

*D*—H⋯*A*	*D*—H	H⋯*A*	*D*⋯*A*	*D*—H⋯*A*
O1*W*—H1*A*⋯O2^i^	0.82	1.99	2.796 (2)	168
O1*W*—H1*B*⋯N2^ii^	0.82	2.33	3.041 (2)	145
C1—H1⋯O2^ii^	0.93	2.42	3.226 (2)	144

Symmetry codes: (i) 
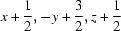
; (ii) 
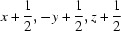
.

**Fig. 1.**
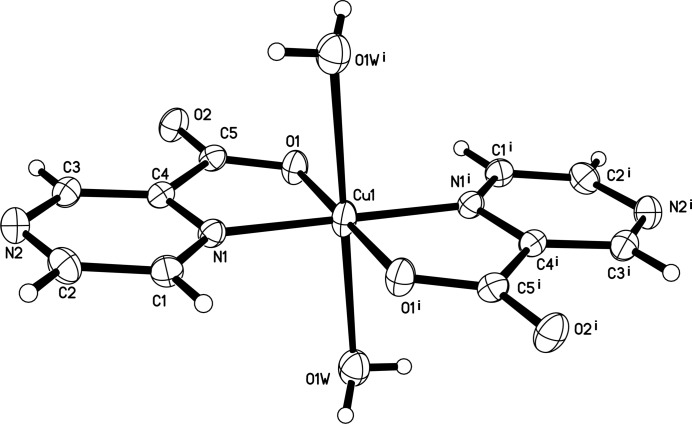
The molecular structure of the title compound. Displacement ellipsoids are drawn at the 30% probability level. [Symmetry code: (i) -x, 1 - y, 2 - z.]

**Fig. 2.**
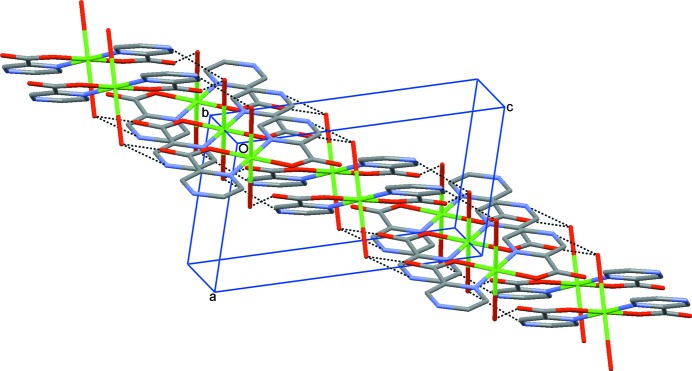
A part of the two-dimensional layer structure in the title compound. Dashed lines denote hydrogen bonds.

## supplementary crystallographic information

### Comment

Pyrazinecarboxylates have been extensively studied as excellent bridging ligands in the coordination chemistry research (Dong *et al.*, 2000; Kubota *et al.*, 2006; Luo *et al.*, 2004; Ptasiewicz-Bak *et al.*, 1995). The structure and magnetic properties of a copper(II) complex with the pyrazine-2-carboxylate (pzc) ligand (polymorph I) has been reported by Klein *et al.* (1982). We report here the structure of a new polymorph (polymorph II) of the title compound.

The polymorph II crystallizes in the monoclinic space group *P*2_1_/*n* (polymorph I in *P*2_1_/*c*). The Cu^II^ atom, lying on an inversion center, is six-coordinated in a distorted octahedral geometry, defined by two O atoms and two N atoms from two pzc ligands in the equatorial plane and two water molecules in the axial positions (Table 1 and Fig. 1). Weak coordination exists between the Cu^II^ center and the coordinated water molecule, with a Cu—O distance of 2.6143 (14) Å, due to Jahn-Teller effects. The bond lengths and angles are in normal ranges (Chutia *et al.*, 2009; Klein *et al.*, 1982). The coordinated water molecule donates its two H atoms to an uncoordinated carboxylate O atom and a pyrazine N atom of the neighboring molecules (Table 2 and Fig. 2). One complex molecule is linked to four neighboring molecules through these O—H···O and O—H···N hydrogen bonds, forming a two-dimensional layer in the (1 0 1) plane. Weak C—H···O hydrogen bond (Table 2) stabilizes the layer structure. In the previously reported polymorph I, the water molecule forms two O—H···O hydrogen bonds with a coordinated carboxylate O atom and an uncoordinated carboxylate O atom. One complex molecule is linked to six neighboring molecules, leading to a three-dimensional network.

### Experimental

Aqueous triethylamine (0.05 ml) was added to a suspending solution of Hpzc (0.012 g, 0.1 mmol) in H_2_O (7 ml), followed by dropwise addition of a solution of Cu(NO_3_)_2_.3H_2_O (0.024 g, 0.1 mmol) in H_2_O (3 ml). The mixture was stirred and sealed in a 15 ml Teflon-lined stainless steel autoclave and heated at 413 K for 3 d under autogenous pressure. When the mixture was cooled to room temperature, blue block crystals of the title compound were obtained (yield 0.029 g, 85% based on Cu).

### Refinement

H atoms of the pyrazine ring were positioned geometrically and refined as riding atoms, with C—H = 0.93 Å and with *U*_iso_(H) = 1.2*U*_eq_(C). H atoms of the water molecule were located in a difference Fourier map and refined as riding, with O—H = 0.82 Å and with *U*_iso_(H) = 1.2*U*_eq_(O).

### Crystal data

**Table:**

[Cu(C_5_H_3_N_2_O_2_)_2_(H_2_O)_2_]	*F*(000) = 350
*M**_r_* = 345.76	*D*_x_ = 1.864 Mg m^−^^3^
Monoclinic, *P*2_1_/*n*	Mo *K*α radiation, λ = 0.71073 Å
Hall symbol: -P 2yn	Cell parameters from 2148 reflections
*a* = 6.7066 (12) Å	θ = 3.1–26.1°
*b* = 7.9041 (14) Å	µ = 1.81 mm^−^^1^
*c* = 12.030 (2) Å	*T* = 293 K
β = 105.036 (2)°	Block, blue
*V* = 615.88 (19) Å^3^	0.29 × 0.25 × 0.20 mm
*Z* = 2	

### Data collection

**Table:**

Bruker SMART APEX CCD diffractometer	1212 independent reflections
Radiation source: sealed tube	1119 reflections with *I* > 2σ(*I*)
graphite	*R*_int_ = 0.014
φ and ω scans	θ_max_ = 26.1°, θ_min_ = 3.1°
Absorption correction: multi-scan (*SADABS*; Sheldrick, 1996)	*h* = −8→8
*T*_min_ = 0.626, *T*_max_ = 0.711	*k* = −8→9
3322 measured reflections	*l* = −7→14

### Refinement

**Table:**

Refinement on *F*^2^	Secondary atom site location: difference Fourier map
Least-squares matrix: full	Hydrogen site location: inferred from neighbouring sites
*R*[*F*^2^ > 2σ(*F*^2^)] = 0.024	H-atom parameters constrained
*wR*(*F*^2^) = 0.071	*w* = 1/[σ^2^(*F*_o_^2^) + (0.0427*P*)^2^ + 0.1736*P*] where *P* = (*F*_o_^2^ + 2*F*_c_^2^)/3
*S* = 1.10	(Δ/σ)_max_ < 0.001
1212 reflections	Δρ_max_ = 0.24 e Å^−^^3^
98 parameters	Δρ_min_ = −0.27 e Å^−^^3^
0 restraints	Extinction correction: *SHELXL97* (Sheldrick, 2008), Fc^*^=kFc[1+0.001xFc^2^λ^3^/sin(2θ)]^-1/4^
Primary atom site location: structure-invariant direct methods	Extinction coefficient: 0.015 (3)

### Fractional atomic coordinates and isotropic or equivalent isotropic displacement parameters (Å^2^)

**Table:**

	*x*	*y*	*z*	*U*_iso_*/*U*_eq_	
Cu1	0.0000	0.5000	1.0000	0.03498 (16)	
O1	−0.0727 (2)	0.61455 (15)	0.85150 (10)	0.0366 (3)	
O2	−0.1196 (2)	0.56084 (18)	0.66400 (11)	0.0410 (3)	
N1	0.0637 (2)	0.30822 (17)	0.90970 (11)	0.0281 (3)	
N2	0.0994 (2)	0.0660 (2)	0.74999 (14)	0.0379 (4)	
C1	0.1310 (3)	0.1525 (2)	0.94293 (15)	0.0323 (4)	
H1	0.1685	0.1252	1.0208	0.039*	
C2	0.1451 (3)	0.0321 (2)	0.86246 (19)	0.0388 (4)	
H2	0.1880	−0.0765	0.8874	0.047*	
C3	0.0352 (3)	0.2226 (2)	0.71834 (15)	0.0334 (4)	
H3	0.0033	0.2509	0.6407	0.040*	
C4	0.0146 (2)	0.3439 (2)	0.79692 (14)	0.0277 (4)	
C5	−0.0665 (3)	0.5207 (2)	0.76592 (16)	0.0299 (4)	
O1W	0.3669 (2)	0.63676 (18)	1.04173 (12)	0.0483 (4)	
H1A	0.3551	0.7207	1.0796	0.058*	
H1B	0.4636	0.5788	1.0781	0.058*	

### Atomic displacement parameters (Å^2^)

**Table:**

	*U*^11^	*U*^22^	*U*^33^	*U*^12^	*U*^13^	*U*^23^
Cu1	0.0529 (2)	0.0279 (2)	0.0235 (2)	0.00894 (12)	0.00891 (15)	−0.00016 (10)
O1	0.0516 (8)	0.0285 (6)	0.0285 (7)	0.0070 (5)	0.0085 (5)	0.0008 (5)
O2	0.0550 (8)	0.0383 (7)	0.0252 (7)	0.0039 (6)	0.0022 (6)	0.0042 (6)
N1	0.0298 (7)	0.0275 (7)	0.0256 (7)	0.0002 (5)	0.0050 (5)	0.0003 (6)
N2	0.0438 (9)	0.0331 (8)	0.0380 (9)	0.0012 (7)	0.0127 (7)	−0.0059 (7)
C1	0.0343 (9)	0.0327 (9)	0.0287 (9)	0.0030 (7)	0.0059 (7)	0.0043 (7)
C2	0.0417 (10)	0.0292 (9)	0.0468 (12)	0.0066 (7)	0.0139 (9)	0.0022 (8)
C3	0.0356 (9)	0.0352 (9)	0.0287 (9)	−0.0023 (7)	0.0073 (7)	−0.0023 (7)
C4	0.0271 (8)	0.0292 (8)	0.0258 (8)	−0.0022 (6)	0.0049 (6)	−0.0005 (6)
C5	0.0299 (8)	0.0287 (8)	0.0292 (10)	−0.0006 (6)	0.0039 (7)	0.0010 (7)
O1W	0.0578 (9)	0.0406 (8)	0.0456 (8)	0.0054 (6)	0.0116 (7)	−0.0037 (6)

### Geometric parameters (Å, °)

**Table:**

Cu1—O1	1.9486 (12)	C1—C2	1.378 (3)
Cu1—N1	1.9753 (14)	C1—H1	0.9300
Cu1—O1W	2.6143 (14)	C2—H2	0.9300
O1—C5	1.279 (2)	C3—C4	1.379 (2)
O2—C5	1.227 (2)	C3—H3	0.9300
N1—C1	1.336 (2)	C4—C5	1.510 (2)
N1—C4	1.340 (2)	O1W—H1A	0.82
N2—C3	1.333 (3)	O1W—H1B	0.82
N2—C2	1.335 (3)		
			
O1^i^—Cu1—O1	180.0	N1—C1—H1	119.8
O1^i^—Cu1—N1^i^	83.73 (5)	C2—C1—H1	119.8
O1—Cu1—N1^i^	96.27 (5)	N2—C2—C1	122.29 (18)
O1^i^—Cu1—N1	96.27 (5)	N2—C2—H2	118.9
O1—Cu1—N1	83.73 (5)	C1—C2—H2	118.9
N1^i^—Cu1—N1	180.0	N2—C3—C4	122.17 (16)
O1W—Cu1—N1	95.50 (5)	N2—C3—H3	118.9
O1W—Cu1—O1	89.03 (5)	C4—C3—H3	118.9
O1W—Cu1—N1^i^	84.50 (5)	N1—C4—C3	120.37 (16)
O1W—Cu1—O1^i^	90.97 (5)	N1—C4—C5	115.01 (15)
C5—O1—Cu1	114.57 (11)	C3—C4—C5	124.60 (16)
C1—N1—C4	118.16 (14)	O2—C5—O1	126.37 (16)
C1—N1—Cu1	130.31 (12)	O2—C5—C4	118.61 (16)
C4—N1—Cu1	111.33 (11)	O1—C5—C4	115.02 (15)
C3—N2—C2	116.61 (16)	H1A—O1W—H1B	109
N1—C1—C2	120.36 (16)		

Symmetry codes: (i) −*x*, −*y*+1, −*z*+2.

### Hydrogen-bond geometry (Å, °)

**Table:**

*D*—H···*A*	*D*—H	H···*A*	*D*···*A*	*D*—H···*A*
O1W—H1A···O2^ii^	0.82	1.99	2.796 (2)	168
O1W—H1B···N2^iii^	0.82	2.33	3.041 (2)	145
C1—H1···O2^iii^	0.93	2.42	3.226 (2)	144

Symmetry codes: (ii) *x*+1/2, −*y*+3/2, *z*+1/2; (iii) *x*+1/2, −*y*+1/2, *z*+1/2.
